# Supplementary material for: Coherent emission from surface Josephson plasmons in striped cuprates
Source: Proc Natl Acad Sci U S A. 2022 Sep 20;119(39):e2211670119. doi: 10.1073/pnas.2211670119 (PMC9522412; doi:10.1073/pnas.2211670119)
Supplement: Supplementary File [file pnas.2211670119.sapp.pdf]

Coherent Emission from Surface Josephson Plasmons in Striped Cuprates

D. Nicoletti[a,*], M. Buzzi[a], M. Fechner[a], P. E. Dolgirev[b], M. H. Michael[a,b], J. B. Curtis[b,c], E. Demler[d], G. D. Gu[e], and A. Cavalleri[a,f]

[a] *Max Planck Institute for the Structure and Dynamics of Matter, 22761 Hamburg, Germany*

[b] *Department of Physics, Harvard University, Cambridge, Massachusetts 02138, USA*

[c] *John A. Paulson School of Engineering and Applied Sciences, Harvard University, Cambridge, MA 02138, USA.*

[d] *Institute for Theoretical Physics, ETH Zurich, 8093 Zurich, Switzerland*

[e] *Condensed Matter Physics and Materials Science Department, Brookhaven National Laboratory, Upton, NY, USA*

[f] *Department of Physics, Clarendon Laboratory, University of Oxford, Oxford OX1 3PU, United Kingdom*

* *e-mail: daniele.nicoletti@mpsd.mpg.de*

Supporting Information

S1. Polarization dependence

S2. Pump pulse length dependence

S3. Fitting model

S4. Equilibrium Josephson plasma frequencies and detection bandwidth

S1. Polarization dependence

In Figure S1 we report a polarization dependent study. Data was taken on $La_{1.905}Ba_{0.095}CuO_4$ at $T = 7$ K after installing a tunable waveplate and an optical polarizer in the pump beam, as well as a THz polarizer in front of the detection crystal. As is evident from the traces, the multi-cycle THz emission signal at $\omega \simeq \omega_{JPR}$ appears to be entirely polarized along the out-of-plane crystallographic axis. Moreover, it is found only upon excitation with pump pulses polarized along the same c-axis direction (see Fig. S1d).

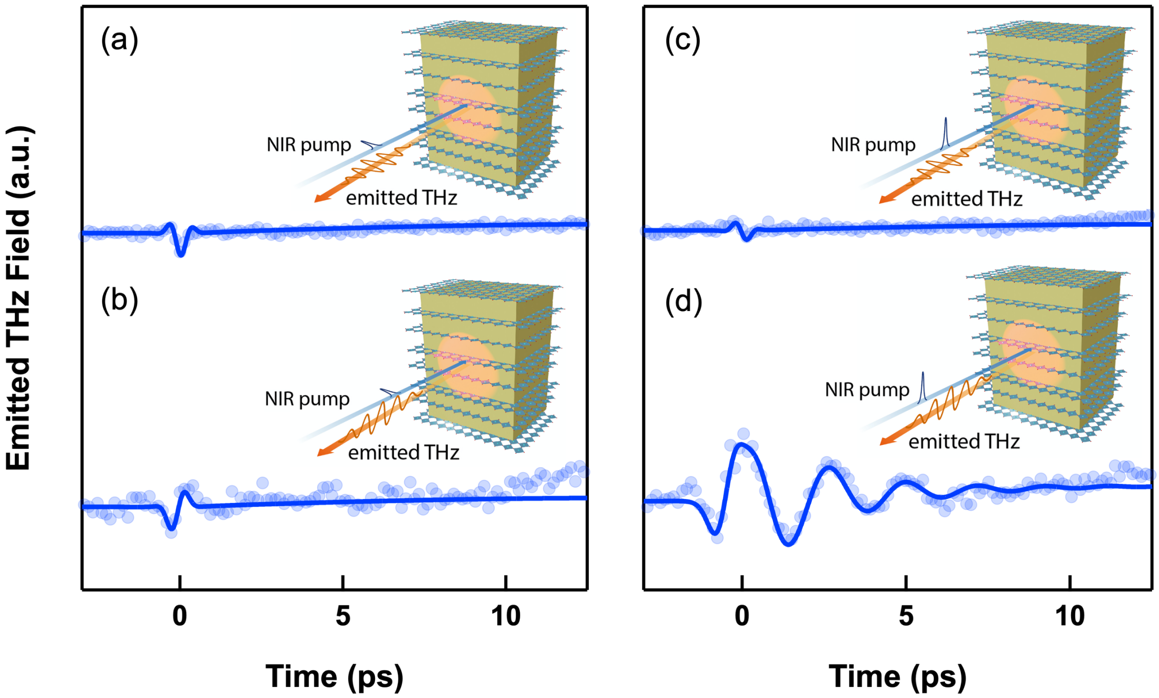

Figure S1. Pump and THz polarization dependence measured in $La_{1.905}Ba_{0.095}CuO_4$ at $T = 7$ K for a pump fluence of 2.5 mJ/cm^2. The following configurations are reported: **(a)** Pump and emitted THz both polarized in-plane, **(b)** Pump in-plane and emitted THz out-of-plane, **(c)** Pump out-of-plane and emitted THz in-plane, **(d)** Pump and probe both polarized out-of-plane. In each panel full circles are the experimental data while solid lines are multi-component fits.

This type of response, consistent with a dipolar distribution, allows us to rule out that the long-lived coherent oscillations originate from higher-order nonlinearities (electric quadrupole or magnetic dipole). These had been identified in Ref. (i) as responsible for the THz emission in unbiased $YBa_2Cu_3O_{6+x}$. Therein, however, the signal had very different characteristics from the one reported here, being present even in the normal state above T_C, and having a single-cycle shape and a 4ϕ dependence with respect to the azimuthal angle ϕ.

S2. Pump pulse length dependence

As discussed in the main text, in a centrosymmetric cuprate impulsive excitation of Josephson plasmons is forbidden by symmetry.

One possibility is that photoexcitation leads to a direct coupling with another higher frequency fully symmetric mode that, in turn, can decay into Josephson plasmons. For example, an amplification of phase modes mediated by the amplitude mode[ii,iii,iv,v] has already been discussed in charge-density-wave materials[vi]. In Fig. S2 we report additional measurements in which we studied how THz emission in LBCO 9.5% evolved as the pump pulses were made longer, at constant fluence and temperature. The emitted oscillation amplitude reduced significantly as the pulse duration exceeded 2 ps [vii], pointing to a scenario in which modes at ~0.5 THz are excited directly, rather than indirectly by a high frequency symmetric mode.

The mechanism proposed in the main text, which involves the direct excitation of surface Josephson plasmons, is compatible with these results.

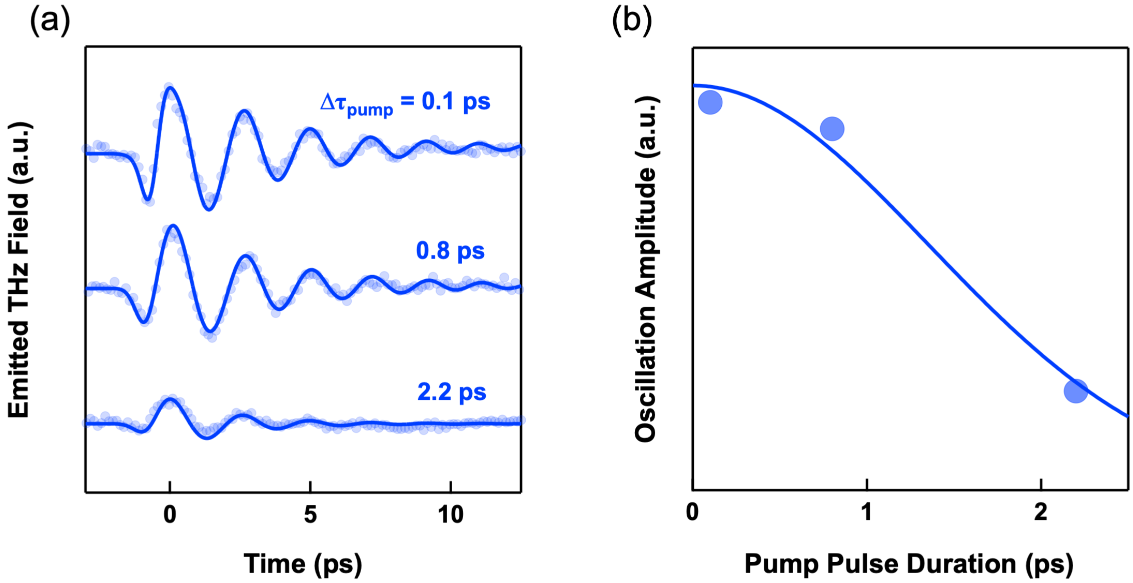

Figure S2. Pump pulse length dependence of the THz emission signal in $La_{1.905}Ba_{0.095}CuO_4$. **(a)** Experimental traces in time domain, taken at for different pump pulse durations, $\Delta\tau_{pump}$ (full circles). All data have been taken at $T\ =\ 7$ K, for a constant pump fluence of 2.5 mJ/cm². Solid lines are multi components fits. **(b)** Pulse length dependence of the THz oscillation amplitude (full circles), extracted from the fits in (a). The solid line is a guide to the eye.

S3. Fitting model

All time-dependent experimental curves shown in Fig. 1 and Fig. 2 of the main text, as well as in Fig. S1 and Fig. S2 of the SI Appendix, were fitted using the formula:

$$E_{THz}(t) = A_0 e^{-(t/2\tau_0)^2}\cos(\omega_0 t + \varphi_0)$$
$$+ A_1[1 + \mathrm{erf}(t/\tau_1)]e^{-\gamma_1 t}\cos[(\omega_1 + c_1 t)t + \varphi_1] + B(t)$$

Here, A_0, τ_0, ω_0, and φ_0 are the amplitude, Gaussian width, central frequency, and phase of the “single-cycle” component around time zero. A_1, τ_1, γ_1, ω_1, c_1 and φ_1 are instead amplitude, rise time, decay rate, initial frequency, linear chirp coefficient, and phase of the quasi-monochromatic, long-lived oscillation. $B(t)$ is a weak, slowly-varying polynomial background.

All fitting curves are displayed in Fig. 1, Fig. 2, Fig. S1, and Fig. S2 as solid lines. Note that for LSCO and LBCO 11.5% the experimental traces were reproduced using only a

“single-cycle” component and weak a slowly-varying background (first and third terms in the equation above). In contrast, for LBCO 15.5% and LBCO 9.5% it was necessary to introduce the multi-cycle oscillation, which typically had much larger amplitude, A_1, than the other terms and only a weak ($\lesssim$ 2%) positive linear chirp. Figure S3a illustrates such a fit for a particular set of data taken on LBCO 9.5%. The individual fit components are explicitly shown.

As reported in Fig. S3b, the “single-cycle” pulse was absent at low fluence and grew quadratically in amplitude with irradiation. On the other hand, the quasi-monochromatic, long-lived oscillation grew linearly up to about 1 mJ/cm^2 and saturated for higher excitation fluence. This linear trend of the main oscillation is compatible with the direct impulsive excitation of a coherent mode.

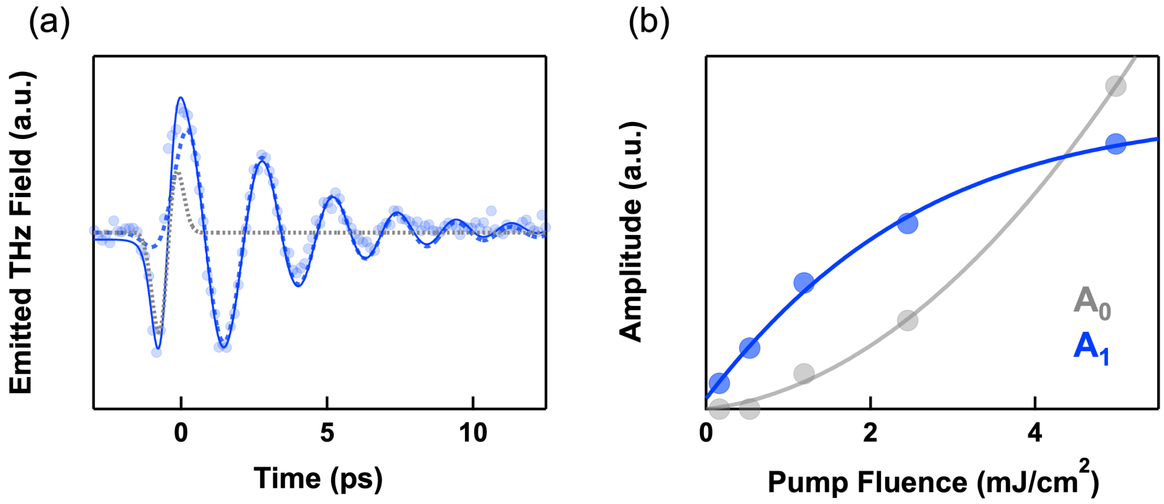

Figure S3. (a) THz emission signal measured in $La_{1.905}Ba_{0.095}CuO_4$ at T = 7 K, for a pump fluence of 5 mJ/cm^2. Full circles are experimental data, while the blue solid line is the result of a multi-component fit. This included a “single-cycle” component around time zero (gray dots) and a quasi-monochromatic, long-lived oscillation (blue dashed line). **(b)** Fluence dependent amplitudes of both single-cycle (gray) and quasi-monochromatic (blue) components, extracted from fits as those shown in panel (a).

S4. Equilibrium Josephson plasma frequencies and detection bandwidth

In Figure S4, we show the temperature dependence of the equilibrium Josephson plasma resonance for all four compounds investigated in our study. The spectra were measured by THz time-domain spectroscopy on the same crystals used in the emission experiment, and are in good agreement with previous reports in the literature[viii,ix,x].

Figure S5 shows instead the results of a measurement performed to estimate the bandwidth of our setup. We have installed a ZnTe crystal, identical to that used for detection of the emission signal, on the cryostat cold finger at the sample position. In Fig. S5a we report the THz electric field emitted by this ZnTe crystal, while Fig. S5b shows the corresponding spectrum. The frequency range shaded in red indicates a plausible detection bandwidth for our setup, estimated by setting the extremes to frequency values for which the spectral amplitude is reduced to 10% of the peak value. This extends over a range of $\sim$0.1 to 2.75 THz, covering the Josephson plasma frequencies of all samples studied, which are indicated by arrows following the color coding of Fig. S4.

We stress here that although putative coherent emission from LBCO 11.5% could have occurred at frequencies lower than $\omega_{JPR} \simeq 0.2$ THz, we found a complete absence of long-lived oscillations in this material for any pump fluence down to values as low as 0.1 mJ/cm^2, for which, based on the LBCO 9.5% results of Fig. 2 and Fig. 3, emission should occur very close to ω_{JPR}.

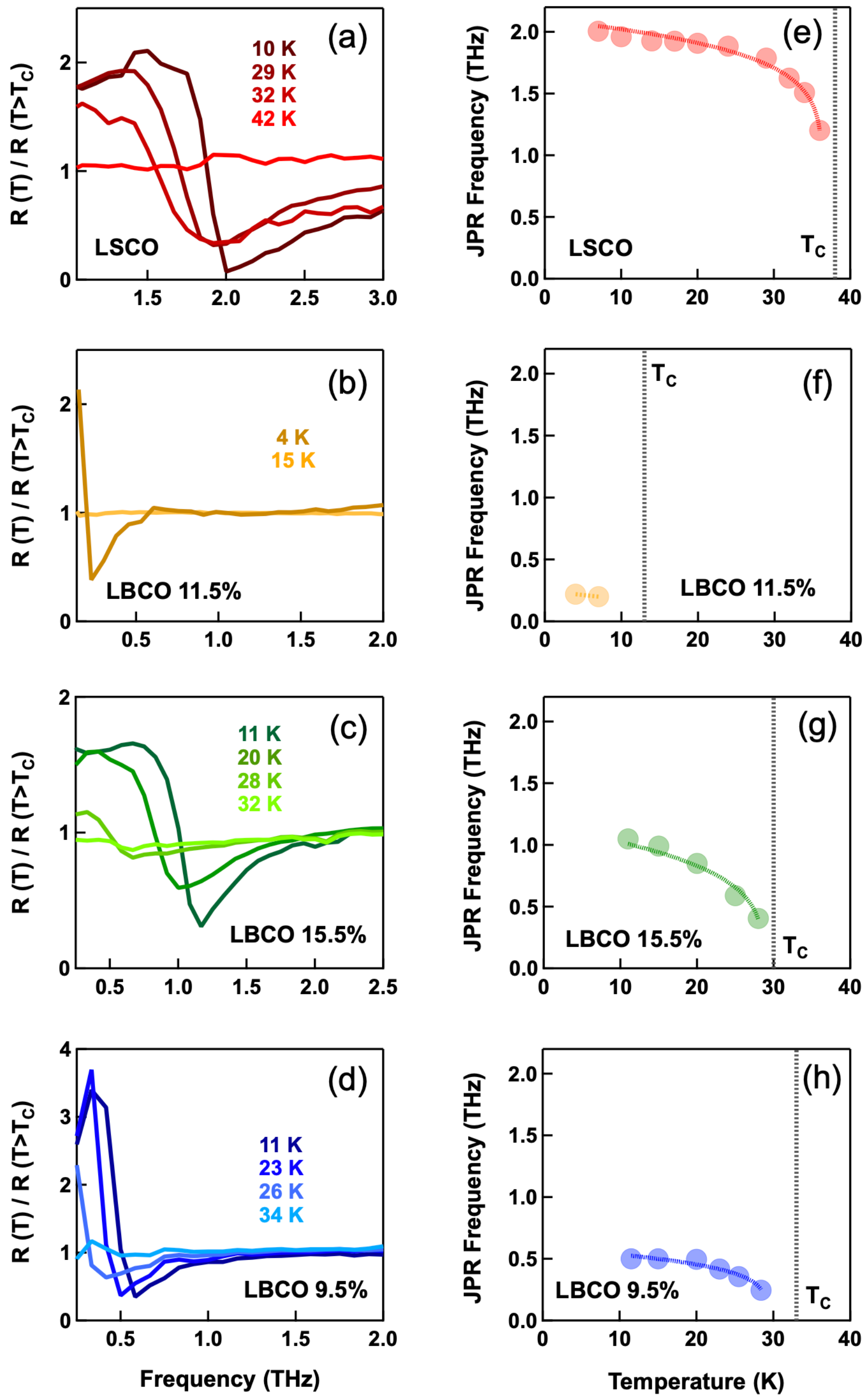

Figure S4. (a-d) Reflectivity of the four samples under study, normalized for the same quantity measured in the normal state above T_C. All spectra were measured by THz time-domain spectroscopy and are reported at selected temperatures below and across the superconducting transition. The reference temperatures for normalization were 50 K (LSCO), 25 K (LBCO 11.5%), and 40 K (LBCO 15.5% and LBCO 9.5%). The Josephson plasma resonance appears here as a sharp edge located at $\omega = \omega_{JPR}$. **(e-h)** Temperature dependence of ω_{JPR} extracted via fits on the reflectivity ratios in (a-d) with a Josephson plasma model (see Fig. 3a). The colored dashed lines are guides to the eye, while the vertical gray lines indicate the superconducting T_C of each sample.

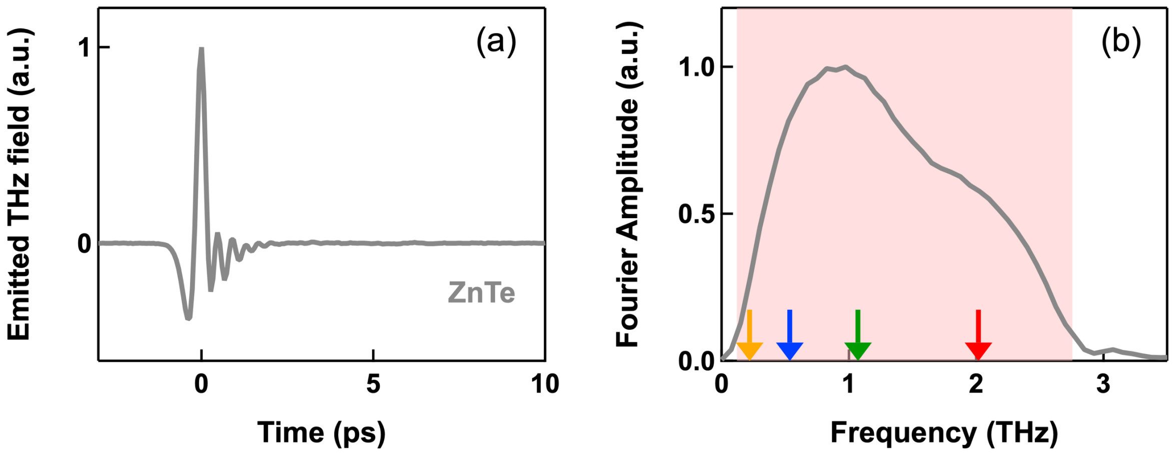

Figure S5. (a) Time-dependent electric field emitted by a 1-mm thick ZnTe crystal mounted on the cryostat cold finger at the sample position. The signal was measured by electro-optic sampling in an identical 1-mm thick ZnTe crystal, which was also used for all other measurements. **(b)** Fourier transform of the time trace in (a). The red shading indicates the spectral range between 0.1 and 2.75 THz, which is an estimate of the bandwidth of our experimental setup, having as extremes the frequencies for which the spectral amplitude is 10% of the peak value. Colored arrows indicate the Josephson plasma frequencies of the four samples in our study extracted at the lowest temperatures shown in Fig. S4. The color coding follows that of Fig. S4.

REFERENCES (Supporting Information)

[i] J. L. W. Siders, S. A. Trugman, F. H. Garzon, R. J. Houlton, A. J. Taylor. *Phys. Rev. B* **61**, 13633 (2000).

[ii] P. B. Littlewood, C. M. Varna. *Phys. Rev. B* **26**, 4883 (1982).

[iii] R. Grasset, T. Cea, Y. Gallais, M. Cazayous, A. Sacuto, L. Cario, L. Benfatto, M.-A. Méasson. *Phys. Rev. B* **97**, 094502 (2018).

[iv] R. Shimano, N. Tsuji. *Annu. Rev. Condens. Matter Phys.* **11**, 103-124 (2020).

[v] B. Mansart, J. Lorenzana, A. Mann, A. Odeh, M. Scarongella, M. Chergui, F. Carbone. *Proc. Natl. Acad. Sci. U.S.A.* **110**, 4539-4544 (2013).

[vi] H. Y. Liu, I. Gierz, J. C. Petersen, S. Kaiser, A. Simoncig, A. L. Cavalieri, C. Cacho, I. C. E. Turcu, E. Springate, F. Frassetto, L. Poletto, S. S. Dhesi, Z.-A. Xu, T. Cuk, R. Merlin, A. Cavalleri. *Phys. Rev. B* **88**, 045104 (2013).

[vii] O. V. Misochko. *J. Exp. Theor. Phys.* **123**, 292-302 (2016).

[viii] S.V. Dordevic, S. Komiya, Y. Ando, D. N. Basov. *Phys. Rev. Lett.* **91**, 167401 (2003).

[ix] C. C. Homes, M. Hücker, Q. Li, Z. J. Xu, J. S. Wen, G. D. Gu, J. M. Tranquada. *Phys. Rev. B* **85**, 134510 (2012).

[x] D. Nicoletti, E. Casandruc, Y. Laplace, V. Khanna, C. R. Hunt, S. Kaiser, S. S. Dhesi, G. D. Gu, J. P. Hill, A. Cavalleri. *Phys. Rev. B* **90**, 100503(R) (2014).
